# Characterization of subtilosin gene in wild type *Bacillus* spp. and possible physiological role

**DOI:** 10.1038/s41598-022-13804-y

**Published:** 2022-06-22

**Authors:** Muaaz Mutaz Alajlani

**Affiliations:** Department of Pharmaceutical Biology/Pharmacognosy, Institute of Pharmacy, University of Halle-Wittenberg, Hoher Weg 8, 06120 Halle (Saale), Germany

**Keywords:** Antimicrobials, Applied microbiology, Bacteria, Microbial communities, Environmental microbiology, Microbial genetics

## Abstract

In a designed study to screen for antimicrobial exhibiting bacteria using molecular aspects, *Bacillus* species were considered to investigate antibiotic biosynthesis genes. 28 bacterial strains and 3 induced mutants were screened for the presence of subtilosin gene (*sbo*) and subtilosin through PCR and Mass spectrometry respectively. *Sbo* gene was detected in 16 out of 28 *Bacillus* strains. The results from gene sequences deliberated by multiple sequence alignments revealed high-level homology to the sequences of the *sbo-alb* gene locus of *B. subtilis* 168 and the other limited reported strains. Hence, this report provided additional strains to support the idea of subtilosin gene predominance amongst *Bacillus* strains isolated from environment and to find different species containing homologous genes, furthermore the utilization of its conserved region as a means of identifying *Bacillus* spp. that produce subtilosin. This is the first report to confirm the detection of subtilosin production from *B. amyloliquefaciens*.

## Introduction

**Figure 1:**
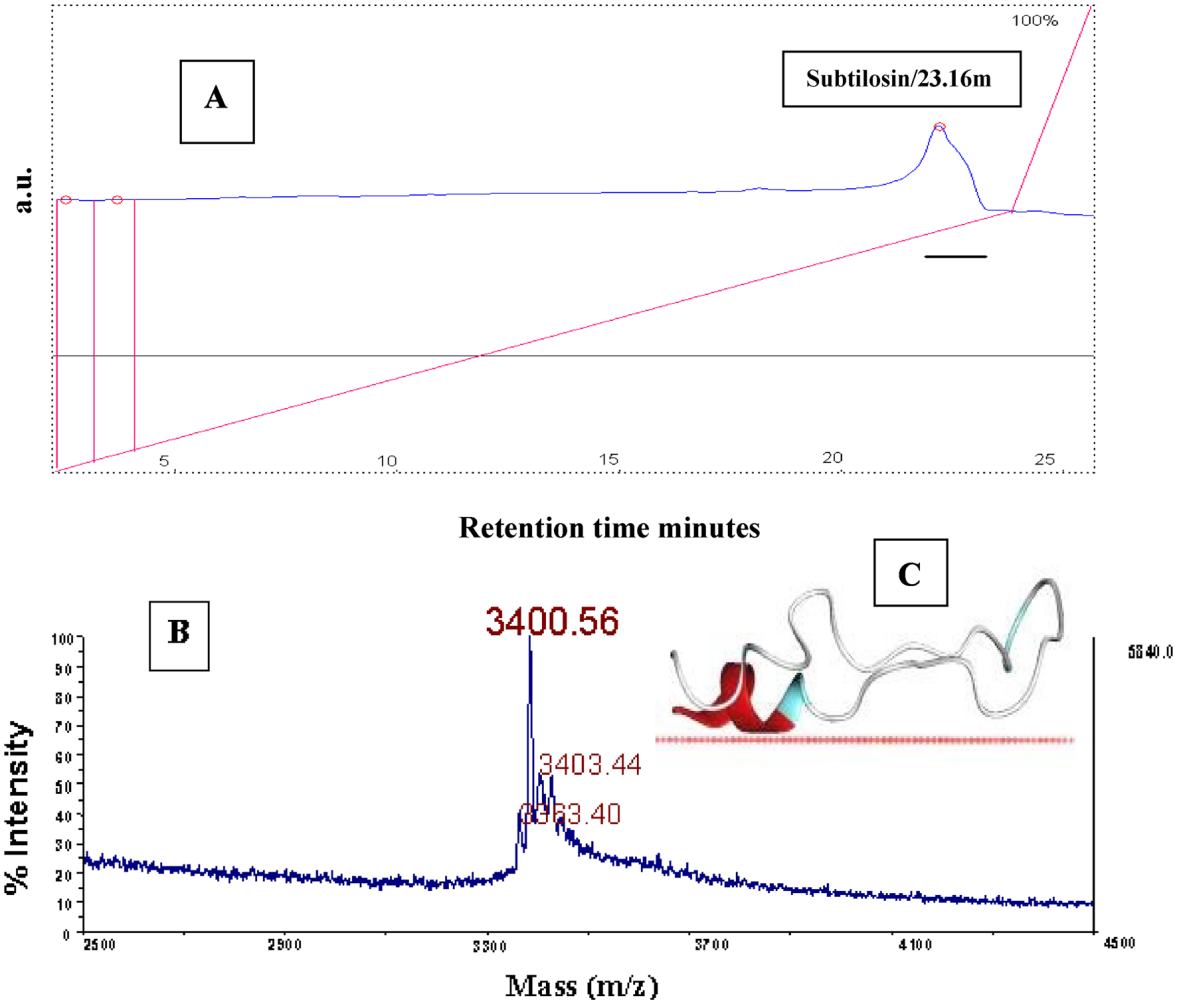
(**A**) RP-HPLC, (**B**) MALDI-TOF-MS, intensive signals correspond to the molecular mass of protonated subtilosin [M + H]^+^ (**C**) 3D structure of subtilosin.

The soil bacterium *Bacillus subtilis* represents bacteria that produce a series of peptide antibiotics^1^. They are members of both classes: the ribosomally synthesized e.g. subtilin^2^, Ericin^3^ and sublancin^4^ and the nonribosomally synthesized such as the lipopeptides surfactin^5,6^, mycosubtilin^7^, and fengycin^8^ Bacilysocin^9^ and 3,3'-Neotrehalosadiamine ^10^. Subtilosin A is one of many antibiotics produced by *Bacillus* strains^11,12^ its importance and role in *Bacillus* group is little understood. Subtilosin is a macrocyclic structure (Fig. 1C) with three inter-residual linkages^13^ that have been elucidated as thioether bonds between cysteine sulphurs and amino acid alpha-carbons^14^. An acidic isoelectric point differentiates subtilosin from the basic lantibiotics^15^. In subtilosin, posttranslational linkage of a thiol to the R-carbon of an amino acid residue is unprecedented in ribosomally synthesized peptides or proteins, and very rare in secondary metabolites^14,16^. The mature product is formed by loss of an unusually short seven amino acid leader peptide, cyclization of the N and C termini, and further modification of Cys, Thr, and Phe residues^17^. The mature subtilosin peptide is highly resistant to enzymatic proteolysis and is stable to moderate heat and acid treatment. It acts against a variety of Gram-positive bacteria, including *Listeria* and other pathogens^18–21^. The production of mature subtilosin is based on the expression of the *sbo-alb* gene cluster encompassing the subtilosin structural gene *sbo* and genes involved in posttranslational modification and processing of presubtilosin and in immunity^22–24^. Expression of the *sbo-alb* genes occurs under stress conditions^25^. 16S rRNA gene used for rapid identification of the *Bacillus* genus was undertaken by Celandroni et al.^26^. The validity of using a hypervariable region (nucleotides 70–344) of the gene was proven adequate to discriminate between all the species except between *B. cereus* and *B. anthracis* and between *B. mojavensis* and *B. atrophaeus*. The high 16S rRNA gene sequence similarities between some stains within this genus can even share phenotypic properties^27^. However, they have been classified as different species based on DNA association values hence, demonstrated the need for a polyphasic approach to the systematics of this genus^28,29^. This was observed between *B. subtilis* subsp. *subtilis* and *B. subtilis* subsp. *spizizenii*, which share phenotypic profiles but have segregated based on DNA reassociation values of 58–69%, in addition to minor polymorphisms in the 16S rRNA gene between the type strains^26,30^. Further, *B. mojavensis* and *B. subtilis* subsp. *spizizenii* have only a 1-bp difference in the 16S rRNA gene and can only be distinguished from each other by sexual isolation, divergence in DNA sequences of the *rpoB* and *gyrA* genes, and fatty acid composition^31^. Stein et al.^32^ pre-postulate the coding gene subtilosin gene (*sbo*) to develop evolutionary divergence in *B. subtilis* subspecies too. This report is to describe subtilosin production by 16 wild-type *B. subtilis* strains and *B. amyloliquefaciens*. The *sbo* genes of these organisms were sequenced in order to analyze the genetic variation between *B. subtilis* wild-type strains. Finally, we confirm the association between production of subtilosin A and the detection of *sbo* gene using PCR screening.

## Materials and methods

### Strains and media

The following 32 different bacterial strains were tested for their sensitivity to the antibiotic using the agar-well diffusion assay: *Bacillus subtilis* 168, *Bacillus subtilis* ATCC*,* from Bacillus Genetic Stock Center (BGSC) and *Bacillus cereus* (Lab. collection) and 28 environmental isolates (Table 1). *Bacillus fusiformis* was routinely used for sensitivity test. All the strains were regularly maintained on nutrient agar , however, for antibiotic production Landy medium (glucose, 20 g L^−1^; glutamic acid, 2 g L^−1^, (NH_4_)_2_SO_4_, 2.3 g L^−1^; yeast extract, 1 g L^−1^; K_2_HPO_4_, 1 g L^−1^; MgSO_4_ 0.5 g L^−1^; KCl, 0.5 g L^−1^; CuSO_4_, 1.6 mg L^−1^; Fe_2_(SO_4_)_3_, 1.2 mg L^−1^; and MnSO_4_, 0.4 mg L^−1^) was used.

**Table 1:** Identification of *Bacillus* strains used through 16S rRNA, *sbo* and biochemical characterization through CHB-50 PCR of. Subtilosin A production was also noted for correlation.

S.n. and Strain designation^a^		Source	Homology 16S r RNA	Sbo PCR detection^b^	Final Bacterial identification	Subtilosin A production^c^
1.	*B. subtilis* 168	BGSC	*B. subtilis* subsp. *subtilis* 168	+I	*B. subtilis* subsp. *subtilis* 168	+
2.	*B. subtilis*	SOIL	*B. subtilis* subsp. *Subtilis*	+I	*B. subtilis* subsp. *subtilis*	+
3.	*B. subtilis*	SOIL	*B. subtilis* subsp. *Subtilis*	+I	*B. subtilis* subsp. *subtilis*	+
4.	*B. subtilis*	SOIL	*B. subtilis* strain CICC148	+I	*B. subtilis* subsp. *subtilis*	+
5.	*B. subtilis*	SOIL	*B. subtilis* subsp. *Subtilis*	+I	*B. subtilis* subsp. *subtilis*	+
6.	*B. subtilis*	SOIL/WATER	*B. subtilis* subsp. *Subtilis*	+I	*B. subtilis* subsp. *subtilis*	+
7.	*B. subtilis*	SOIL/WATER	*B. subtilis* subsp. *Subtilis*	+I	*B. subtilis* subsp. *subtilis*	+
8.	*B. subtilis*	SOIL	*B. polyfermenticus*	+I	*B. subtilis* subsp. *subtilis*	+
9.	*B. subtilis*	SOIL	*B. subtilis* subsp. *Subtilis*	+I	*B. subtilis* subsp. *subtilis*	+
10.	*B. fusiformis*	SOIL	*B. sp .*strain MHS006	–	*B. subtilis* subsp. *subtilis*	–
11.	*B. subtilis* 7 mut (-A)	SOIL	*B. subtilis* subsp. *Subtilis*	–	*B. subtilis* subsp. *subtilis*	–
12.	*B. subtilis* ATCC6633	BGSC	*B. subtilis* subsp. *subtilis* ATCC6633	+I	*B. subtilis* subsp. *subtilis* ATCC6633	+
13.	*B. subtilis*	SOIL/DRY	*B. subtilis* subsp. *Spizezinii*	+II	*B. subtilis* subsp. *spizezinii*	+
14.	*B. subtilis*	SOIL/DRY	*B. subtilis* subsp. *Spizezinii*	+II	*B. subtilis* subsp. *spizezinii*	+
15.	*B. mojavensis*	SOIL/DRY	*B. subtilis* strain Au53	+II	*B. subtilis* subsp. *spizezinii*	+
16.	*B. mojavensis*	SOIL	*B. mojavensis*	+I	*B. subtilis*	+
17.	*B. licheniformis*	SOIL	*B. mojavensis*	–	*B. licheniformis*	–
18.	*B. licheniformis*	SOIL	*B. licheniformis*	–	*B. licheniformis*	–
19.	*B. licheniformis*	SOIL	*B. licheniformis*	–	*B. licheniformis*	–
20.	*B. licheniformis*	SOIL	*B. licheniformis*	–	*B. licheniformis*	–
21.	*B. licheniformis*	SOIL	*B. sp.* Ni 21	–	*B. licheniformis*	–
22.	*B. licheniformis*	SOIL	*B. amyloliquefaciens*	–	*B. licheniformis*	–
23.	*B. licheniformis*	SOIL	*B. amyloliquefaciens*	–	*B. licheniformis*	–
24.	*B. licheniformis*	SOIL	*B. licheniformis* strain KL-176	–	*B. licheniformis*	–
25.	*B. licheniformis*	SOIL/DRY	*B. amyloliquefaciens*	–	*B. licheniformis*	–
26.	*B. amyloliquefaciens*	SOIL/DRY	*B. amyloliquefaciens*	+ I	*B. amyloliquefaciens*	+
27.	*B. amyloliquefaciens*	SOIL	*B. amyloliquefaciens*	+ I	*B. amyloliquefaciens*	+
28.	*B. amyloliquefaciens*	SOIL	*B. amyloliquefaciens*	–	*B. licheniformis*	–
29.	*B. amyloliquefaciens*	SOIL/DRY	*B. fusiformis*	–	*B. licheniformis*	–
30.	*B. pumilus*	SOIL	*B. pumilus*	–	*B. licheniformis*	–
31.	*B. amyloliquefaciens*	SOIL/WATER	*B. fusiformis*	–	*B. licheniformis*	–

^a^Identification by using CHB-50.

^b^+ PCR product of the expected size was detected. I and II assigned for subsp. *subtilis* and *spizizenii* respectively.

### Bacterial identification

Identification of the isolated strains was carried on by sequence homology of 16S rDNA accompanied by morphological and biochemical characterization. Identification to the species level was defined as a 16S rDNA sequence similarity of ≥ 99% with that of the prototype strain sequence in GenBank; identification at the genus level was defined as a 16S rDNA sequence similarity of ≥ 97% with that of the prototype strain sequence in GenBank. The biochemical profile of test isolates was determined with the API 50 CHB strips following the manufacturer’s instructions (bioMerieux, France). This test allows bacterial strains to be classified according to their ability to ferment 49 different carbohydrates. The results were analyzed with the APILAB Plus software (bioMerieux, France).

### Antibiotic assay

Samples of culture supernatant containing the antibiotic checked for activity using an agar- well diffusion assay^33^. Fifty µL of *Bacillus fusiformis* liquid culture of 0.3 OD_600_ was spread onto the surface of Petri dish containing L-agar. 50 µL antibiotic sample was transferred into the well made in media plates using a sterile cork borer. The sample was allowed to diffuse into the agar and the plate was inverted and incubated at 37 °C until a lawn of the indicator bacteria appeared on the plate (approximately 10–16 h).

### DNA isolation, extraction and PCR

Genomic DNA extracted from overnight-inoculated bacterial culture in N-broth at 37 °C with 120 rpm. The extraction carried out using gene extraction kit (Biorad). PCR amplification of ~ 1375-bp consisted of *sbo* and flanking region was performed successfully with TS13C (GAATTGACACTATCTAGAGAAATGCCG) and TS14 (ATCCGGTGGTGCGGAATTCGATGA)^32^. While primers 27f. (GAATTGACACTATCTAGAGAAATGCCG) and 1522r (ATCCGGTGGTGCGGAATTCGATGA)^34^ were used to amplify the 16S rRNA gene. Both sets of primers purchased from Gene Link Inc., USA. 0.5–0.1 ng of chromosomal template DNA and 0.25 µM each primer were added to Master mix (Fermantas). The PCR conditions started with heating at 94 °C for 5 min and passed through 30 cycles as follows: denaturation for 30 s at 94 °C primer annealation for 30 s at 59 °C and extension at 72 °C for 1.2 min. The final extension was at 72 °C for 3 min. PCR fragments were excised from agarose gel electrophoresis followed by extraction with a QIAgen gel extraction kit (Qiagen). The isolated PCR products were sequenced by using an ABI Prism dye terminator cycle sequencing ready reaction kit and an ABI PRISM 377 DNA sequencer (Applied Biosystems). Analyses of DNA sequences were performed by using Prochromas version software (Oxford Molecular, UK).

### Production of (-A) No activity mutants

Mutants from strain-7 a confirmed producer of subtilosin were generated from exposed culture to UV light for different intervals. The surviving bacteria were screened for the disruptions in *sbo* gene through PCR and subtilosin production by MALDI-TOF–MS.

### TLC, SPE and RP-HPLC

Bacterial supernatant, recovered by 15,000×*g* centrifugation for 20 min of 36 h old shaken culture were screened for the presence of subtilosin A. Supernatants were extracted with same amount ethyl acetate (Fishers, USA) and vacuum dried. Residues were dissolved in minimal amount of acidified 20% acetonitrile v/v. Further isolation was performed through SPE columns (CHROMABOND C18ec) purchased from Macherey–Nagel, Germany. First columns were preconditioned with methanol then water and samples were applied under low pressure. The columns were washed with water and four fractions were eluted by consecutive four solutions 40, 60, 80 and 100% acetonitrile. Fractions eluted with 80% were accommodated over short lines in TLC sheets (Merck, Germany) and developed with 1:1:1 v/v/v of n-hexane, chloroform and methanol as mobile phase. The plates were analyzed under UV-Light, however, active fractions were detected using narrow strips of developed sheets by a bioassay method with a sensitive test organism^33^. These active fractions were scrapped from the TLC plate and extracted with eluent A (0.1% (vol/vol) trifluoroacetic acid and 20% (vol/vol) acetonitrile). Finally, analysis was by reversed-phase HPLC using Thermo Hypersil-Keystone ODS (particle size, 5 µm; column dimensions, 250 by 4.6 mm, Thermo Hypersil, USA). A sample was applied with eluent A and eluted with segmented gradients of eluent B (0.1% (vol/vol) trifluoroacetic acid and 80% (vol/vol) acetonitrile) as follow 40% eluent B for 30 min and 40–100% eluent B for 10 min. Eluents A and B were composed in Milli-Q HPLC grad water.

### MALDI-TOF-mass spectrometry

Fractions correlated with Subtilosin A from TLC and Reverse phase HPLC were analyzed using MALDI-TOF–MS. 2 µL of sample mixed with 2 µL matrix solution (2 mg of alpha- hydroxycinnaminic acid per ml in acetonitrile-methanol–water (1:1:1) on the target plate. MALDI-TOF–MS spectra were recorded by using a 337-nm nitrogen laser for desorption and ionization. The mass spectrometer operated in the linear mode at an accelerating voltage of 18 kV with an ion flight path that was 0.7 m long. The delay time was 375 ns. Matrix suppression was also used, and the mass spectra were averaged over 50–100 individual laser shots. The laser intensity was set just above the threshold for ion production. External calibration was performed by using the [M + H]^+^ signals of renin, adenocorticotropic hormone, insulin oxidized B, and bovine insulin (Sigma-Aldrich Co.) the results were anticipitated as subtilosin A with m/z of 3400.7 and 3406.6. The variance of the *m/z* of ±0.8 Da was considered acceptable.

## Results

### Identification of bacterial strains

Strains were identified according to their morphological and biochemical characteristics added by homology to 16S rRNA with the type strains available in NCBI and RIDOM^35^ the results revealed the distinction between two subsp. *subtilis* and *spizizenii* as well as their association with their sources.

### Detection and sequencing of sbo gene

**Figure 2:**
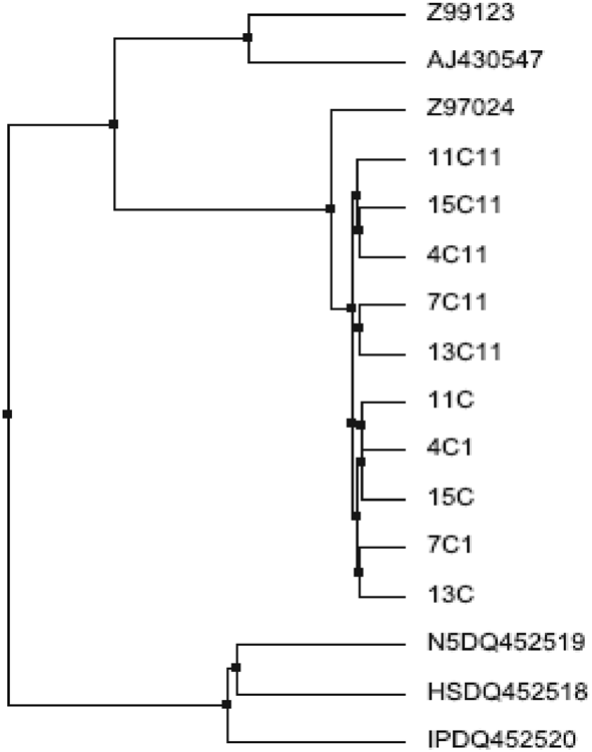
Phylogenetic diversity of isolated strains using multiple sequence alignments of sequences *sbo* and flanking region.

*Sbo* and its flanking region were detected from the environmental strain and Type strains. *B. subtilis* 168 and *B. subtilis* ATCC 6633 were used as positive control representing the two classes/subsp. *subtilis* (class I) and *spizizenii* (class II) respectively. Results are shown in (Table 1) in which majority of strain designated as *B. subtilis* were secured *sbo* class I. All the obtained DNA fragments were sequenced, and the phylogenetic relation was established using Clustal W, EBI (Fig. 2).

### Detection and isolation and of Subtilosin

Subtilosin presence was regularly checked on TLC in reference to match the subtilosin produced by *B. subtilis* 168 further confirmation was carried on using reverse-phase HPLC (Fig. 1A) and MALDI-TOF-MS (Fig. 1B; Table 1).

### Mutation analysis

**Figure 3:**
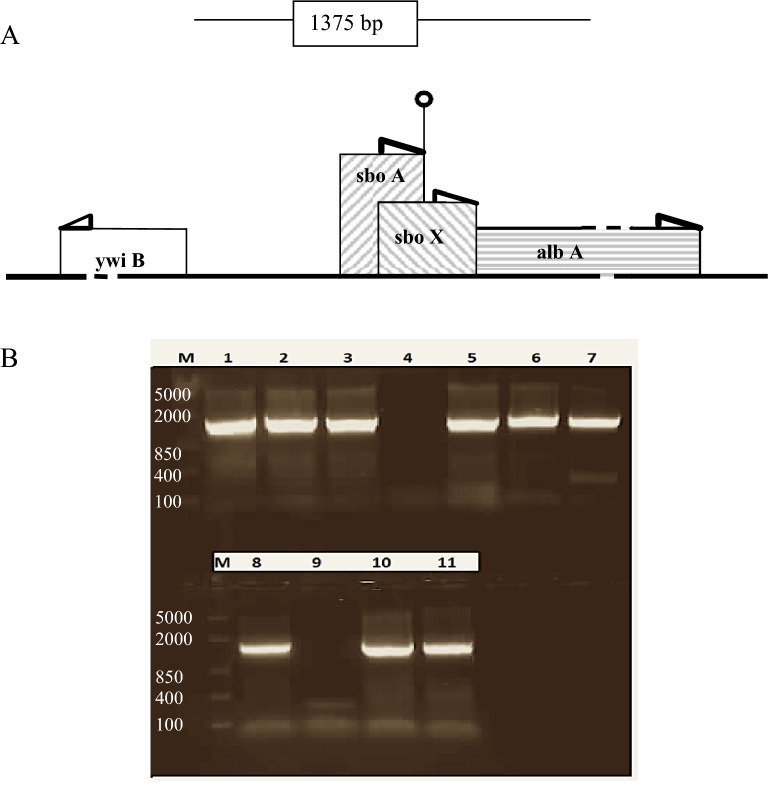
(**A**) Organization of the *sboAX* locus. the *sboA*, *sboX*, and *albA* region of the *sbo-alb* gene cluster and flanking region. Arrowheads indicate direction of transcription; (o) is a terminator. (**B**) PCR amplified fragment of 1375 bp related to *sbo* gene where M indicates marker (5000, 2000, 850, 400, 100 bp). Positive amplification for lanes 1–3, 5–7 corresponding to different *B. subtilis* isolates while lane 10 and 11 coorsponds to *B. amyloliquefaciens* and *B. subtilis* ATCC6633 respectively. Lane 8 is a positive control (*B. subtilis* 168) and Lane 9 is a negative control (*B. licheniformis*).

Mutants produced further selected based on inhibitory activity. Out of 200 mutant 1 strain was isolated with no detectable zone of inhibition. This (-A) strain was not able to produce subtilosin more over the *sbo* was not amplified using PCR. Hence, it was determined to be functional disruption of encoding gene (Fig. 3B).

### Nucleotide sequence accession number

The nucleotide sequences reported deposited in NCBI GenBank under accession numbers; FJ151503, FJ151504, FJ151505, FJ151506, FJ151507.

## Discussion

It is well established that, the ribosomal peptide antibiotics are synthesized during active growth, while nonribosomal ones are synthesized at later stages. Theories on the role of antibiotic production are yet to be investigated. The best-accepted theory is that nonribosomal antibiotics may play a role in competition with other microorganisms during the starvation phase or spore germination^36–38^. While the role of ribosomal peptides remained undefined. Not so obvious the role of such products in the active life cycle. For example the sublancin gene cluster is not essential for *B. subtilis*, however, it contains yet unidentified genes mediating resistance against sublancin action. Suggestions of an intrinsic mechanism of gene improvement i.e. utilizing antibiotics as first line of defense for survival rather than a second, question antibiotics as secondary metabolites. Another probability is displayed social behaviors as co-ordinate gene expression and group behavior through different quorum-sensing pathways^39^. It was determined that interaction of subtilosin with the lipid head group region of bilayer membranes in a concentration dependent manner induced a conformational change in the lipid headgroup and disordering in the hydrophobic region of bilayers that ultimately resulted in membrane permeabilization at high peptide concentrations^40^. Such adoption may lead to assume a growth control during prosperous stage. Furthermore, under anaerobic conditions an increased by 4- to 90-fold, anticipated that the cell accumulates inactive precursors of subtilosin, which then undergo oxygen-dependent modifications to yield an active peptide when an aerobic environment is encountered^41^. The widespread occurrence of subtilosin might reflect an important physiological role. A specific function of subtilosin as an antibiotic, killing factor^42^ or as a pheromone during anaerobiosis^43^ or biofilm growth of *B. subtilis*^39^ could be well thought-out. Never the less the gene encoding subtilosin production has demonstrated a strong biomarker for *Bacillus subtilis*. The *B*. *subtilis* strains have segregated into two subclades, one encompassing strain 168 and the other W23, classified strain 168 as *B. subtilis* subsp. *subtilis* and W23-related strains as *B. subtilis* subsp. *spizizenii* based on DNA reassociation studies^31^ and sbo gene analysis^32^. The W23 and 168 group strains are identical for most phenotypic characteristics. However, cell wall chemistry of the W23 strains and 168 strains were different; the cell wall of the former contained ribitol and glycerol teichoic acids and that of the latter only glycerol teichoic acid^44^. The *sbo*-gene of *B. subtilis* encodes the 43-aminoacid residue comprising the prepropeptide of subtilosin^23^. The nucleotide sequences of the *sbo* genes and flanking regions are identical in strains belonging to the same subspecies, and the sequences differ by three nucleotides in the two subspeciesm^45^. However, the encoded Sbo prepeptides are identical in all cases^32^. On the other hand *sboX*, encoded a bacteriocin-like product, a new gene with an unknown function, in strain 168^23^**,** which resides in an open reading frame overlapping the coding region of *sbo* (Fig. 3A). Notably, the expression of *sboX* would result in a 22-amino-acid curtailed peptide in W23- like strains compared to the peptide produced by 168-like strains, which makes it unlikely that *sboX* is produced by W23-like strains. These observations were further elaborated to support and to evaluate possible evolutionary relationships among the subtilosin producers, however, A correlation between sbo gene and subtilosin production was not established probably due influence of *sboX*,. Previous attempts for *sboX* insertions were not successful as such insertions might render *sboA* mRNA unstable and explain the reduced subtilosin production in *sboX* mutant. Alignments has revealed that the sbo genes is highly conserved with those of *B. subtilis* subsp. *subtilis* (96–100% amino acid identity), while the remaining were less conserved (83–88% identity). This high and low level of conservation is unprecedented too; for example, thymidylate synthases A (*thyA*) in *B. subtilis* subsp. *spizizenii* ATCC 6633 and W23 and *B. subtilis* subsp. *subtilis* (168) exhibit more than 95% amino acid identity^46^. Even the average level of amino acid identity for the DNA gyrases (*gyrA*) in seven *Bacillus* type strains was 95.1%^28^. With these results, we can confirm subtilosin gene predominance amongst *Bacillus* strains isolated from environment and the correlation amongst different sub-species containing homologous genes. Furthermore, this article demonstrated the possibility to utilize *Sbo* conserved region as a mean of identifying *Bacillus* spp. that produce subtilosin.
